# On variability & human consciousness

**DOI:** 10.1016/j.heliyon.2018.e00905

**Published:** 2018-11-01

**Authors:** Brian B. Boutwell

**Affiliations:** a Saint Louis University, College for Public Health and Social Justice, Criminology & Criminal Justice, Department of Epidemiology (Secondary Appointment), United States; b Department of Family & Community Medicine, School of Medicine (Secondary Appointment), United States

**Keywords:** Psychology

## Abstract

The topic of consciousness remains central across numerous academic fields ranging from philosophy to cognitive neuroscience. Scholars in all of these fields continue to debate the origins of conscious experiences. More recently, scientists have applied advanced imaging techniques to illuminate brain regions that are at least associated with our subjective feelings of conscious experience. Though much disagreement remains, one point that is generally accepted across fields is that consciousness is not the product of an immaterial substance, but rather is produced by functioning across physical substrates in the brain. This point of agreement is enough to suggest that genetically and environmentally underpinned individual variation in brain structure may contribute to individual variation in consciousness. To the extent that this is correct, it may provide insight on a host of important questions across various academic fields. Equally important, understanding sources of variability in consciousness may be a key piece of the puzzle for understanding not only how consciousness evolved but also how selection pressures might continue to act on the human experience of consciousness across subsequent generations.

## 1 Introduction

There is no shortage of work discussing human conscious experience. For centuries, philosophers have debated the nature of consciousness, sharply disagreeing with one other at times, yet reaching a consensus regarding the difficult nature of problem (Bor, 2012; Chalmers, 1995; Crick & Koch, 1990, 1998; Dennett, 1991, 2001; Graziano and Kastner, 2011; Kaufman, 2013; Nagel, 1974). In articulating that difficulty, Crick and Koch, note (2003; p. 119): “The most difficult aspect of consciousness is the so-called ‘hard problem’ of qualia^1,2^— the redness of red, the painfulness of pain, and so on. No one has produced any plausible explanation as to how the experience of the redness of red could arise from the actions of the brain.” Indeed, this remains the case currently, yet with the continued progression of scientific inquiry, certain aspects of the consciousness problem seem to be more tractable (Bor, 2012; Crick and Koch, 1998, 2003). Slowly, we have discarded previous ideas about consciousness, thinning the herd of explanations for what it is, and where it comes from (Pinker, 1997). As was noted years ago, there is no “ghost in the machine” (Bor, 2012; Pinker, 2002). We know this because we are capable of altering consciousness by first altering structure or functioning in the brain. Consciousness, regardless of how one defines it, is tethered directly to brain functioning.

The implications of a brain-based consciousness allow us to make several logical inferences about the nature of conscious experience. First, consciousness (somehow) arises from a physical substrate—the brain—that is comprised of tissues produced by coordinated genetic expression. Second, human brains were designed by differential survival and reproduction over the history of our species, which is to say, “designed” by evolutionary forces (Dawkins, 1976). Third, natural selection works on the level of genetic variation utilizing the selective retention of genetic variants that are advantageous to the fitness of the individual (Dawkins, 1976; Williams, 1966). Fourth, because consciousness arises in the brain, it is reasonable to suspect that over the course of evolutionary history, our brains diverged from those of our ancestors in such a way as to experience a different “form” of consciousness compared to our human and non-human ancestors (more on the consciousness levels below). Fifth, this variation is likely to be at least partly the result of genetic variation. Sixth, consciousness varies in our species. Seventh, and finally, brain structure and function is partly heritable. As a result, consciousness may vary naturally in the population for both environmental and genetic reasons. Consciousness—like virtually every other quantifiable human trait—accords with the laws of behavior genetics (Chabris et al., 2015; Turkheimer, 2000).

### 1.1 The goal of the current discussion

Prior to progressing further, it is important to distinguish what the goals of this discussion are, and what they are not. Simply put, I am asserting that consciousness is variable, and perhaps partly heritable. Yet, there are different ways in which to define consciousness, and in the past, some scholars have argued against forcing a particular definition, as it would almost certainly prove premature, incomplete, or in some other way, unsatisfactory (Crick and Koch, 1990). Although this is likely true, it is nonetheless helpful to have some way in which to operationalize the concept of consciousness for the current study. It would seem that relying on a broad conceptualization is helpful, and along those lines we might define consciousness—simply for this particular discussion—as “subjective experience” (Tononi and Koch, 2015; Webb and Graziano, 2015). As Koch and colleagues note (2016; p.307): “Being conscious means that one is having an experience — the subjective, phenomenal ‘what it is like’ to see an image, hear a sound, think a thought or feel an emotion.” Utilizing “subjective experience” also permits the current discussion to have some traction at later points when covering issues of measurement. In particular, it recognizes a reality that experimental scientists are already well aware of, which is that measuring consciousness often means measuring narrower aspects of it (pain sensation, etc).

Equally important, however, is to outline what this paper is not intended to represent. This is not intended to be a theory of how consciousness evolved, or whether it exists as a biological adaptation, or a by-product of some other system of adaptations (Buss, 2009; Polger and Flanagan, 2002; Miller, 1999). It is difficult to imagine a scenario, however, in which clarifying the sources of variation for consciousness fails to reveal anything about its origins, as these topics are relevant for evolutionary discussions of other complex traits (Penke et al., 2007). As others have rightly pointed out, the heritability (or lack thereof) of consciousness is a key point to ponder when discussing the evolution of conscious experience and whether it constitutes an “adaptation” in the strict biological definition of the term (Dennett, 1991; Polger and Flanagan, 2002; Miller, 1999). However, that is not a primary part of the current discussion.

Finally, the purpose of this article is not to propose an answer to what Chalmers (1995) refers to as the “hard problem of consciousness” which is to say, why we have subjective experience in the first place (see also, Nagel, 1974). The issue of “qualia” and the “redness of red” is fascinating, but not something this review can resolve (Crick and Koch, 2003). Rather, what I address is decidedly in the realm of the “easy” problem, as it bears directly on why brains vary from person to person, and thus why consciousness (subjective experience)—a product of brain activity—might also vary (for reasons other than purely environmental factors). With that in mind, it is key to make a further distinction about consciousness.

### 1.2 Consciousness: levels & contents

Tononi and Koch (2008), as well as others, have pointed out the distinction regarding levels of consciousness versus the content of consciousness. To discuss levels of consciousness, in and of itself, is to acknowledge that consciousness can vary across time and across individuals in a population. For instance, falling asleep is indicative of a diminishing of conscious awareness (Bor, 2012; Tononi and Koch, 2008). As individuals progress toward sleep, they continually lose awareness until reaching a state of consciousness far diminished from that which is experienced when awake. Another example is both entering and exiting general anesthesia, thus traversing “levels” of conscious awareness (Bor, 2012; Tononi and Koch, 2008; Långsjö et al., 2012). As one succumbs to the influence of the drugs, awareness rapidly creeps away. Once the drugs subside, an individual seemingly returns from unconsciousness, becoming increasingly aware of their surroundings.

Of course, it is incorrect to suggest that the individual is “returning” from anywhere. Despite the intuitive appeal of the phrase, what has taken place is the return of appropriate functioning in the central nervous system, such that consciousness once again arises from physical substrate (Bor, 2012; Långsjö et al., 2012). In the case of sleep, brain function is altered by physiological processes, whereas general anesthesia utilizes drugs to alter levels of consciousness. In both cases, however, the point of the current discussion is illustrated quite directly, which is that consciousness can vary for both environmental and even biological reasons. Before progressing, however, it is worth making an additional general point, which is that just because levels of consciousness can be varied by environmental factors (i.e., anesthesia), this does *not* represent prima facea evidence that variation across levels of consciousness is also partly heritable (a point which will be returned to momentarily).

The content of consciousness, on the other hand, refers to the experience one has in a given instance. The now famous thought experiment and essay of “What is it like to be a bat” is one example in that, assuming it is “like something” to be a bat, one can reasonably conclude that both the bat is conscious, and its consciousness has some sort of content (Nagel, 1974). The content of consciousness is both important and interesting. Moreover, it should not be considered as totally divorced from levels of consciousness. At the very least, the *level* at which one is conscious should inform the *content* of what one is capable of experiencing (Tononi and Koch, 2008). The content of one's experience when emerging from anesthesia is bound to be different than when one is completely awake and eating dinner.

Nonetheless, there are two somewhat distinct, yet also interlocking questions to be dealt with. First, would the “average” level of human consciousness be expected to vary naturally in a population? Second, would the content of conscious experience be expected to vary naturally in a population of individuals? If the answer to either, or both, questions is “yes” then it becomes reasonable to discuss sources of variation (both genetic and environmental). It is possible, too, that the answer to either question may well be different, so too may the sources of variation differ. Levels of consciousness (assuming they vary), may differ for reasons related largely to environmental variation, while contents of consciousness may differ for reasons related to both genetic and environmental variation. These issues arise at multiple points below, and follow a brief primer on how quantitative geneticists think about sources of variation in a population of organisms, humans included.

## 2 Background

### 2.1 A primer on behavior genetics

Quantitative genetics is a research field that relies primarily on the analysis of sibling and family data (i.e., monozygotic twins, dizygotic twins, full siblings, etc.) in order to decompose phenotypic variance into that which is explained by genetic differences in a population, from that which is explained by environmental differences across members of a population (Plomin et al., 2013). The techniques of quantitative genetics have proven useful for examining the sources of variance for not only disease and psychopathology (e.g., depression, schizophrenia), but also the normally distributed quantitative traits studied widely in the psychological sciences such as personality styles and general intelligence (Polderman et al., 2015; Sullivan et al., 2003).

Extended reviews regarding the theoretical and mathematical assumptions of quantitative genetics are available (Plomin et al., 2013; Barnes et al., 2014). Briefly, researchers employing the techniques of quantitative genetics—among other goals—seek to divide trait variance into three categories: heritability, shared environment, and non-shared environment. Heritability captures trait variance owing to genetic variation, shared environment captures trait variance owing to family level processes which function to make siblings raised together similar to one another, and non-shared environment captures all environmental processes that create differences between siblings, as well as all measurement error in the model (Barnes et al., 2014).

Calculating each of these parameters in an unbiased fashion hinges on satisfying certain assumptions in the model, the two most prominent of which are the equal environments assumption, as well as the assumption of random mating. As prior work has pointed out, these assumptions are sometimes, even often, violated, especially random mating (Barnes et al., 2014). Yet, this same work demonstrated that the bias resulting from such assumption violations is in general small, and does not alter the substantive conclusions gleaned from quantitative genetics work. Put differently, parameter estimates for heritability, as well as shared and non-shared environment can be considered generally robust and reliable even in the presence of violated assumptions.

Almost two decades ago, Turkheimer (2000) presented what would become a seminal paper on the topic of behavioral genetics and the research conducted up to that point. In particular, Turkheimer (2000) suggested that the weight of evidence was at a point in which “laws” could be derived concerning why most quantitative traits vary in the population. By laws, Turkheimer (2000) referred of course to very general principles which suggested that: 1) all traits are, to varying degrees, heritable; 2) the impact of the shared environment on variation is less than that of genes; 3) the impact of the non-shared environment is consistently important for understanding human variation and 4) (recently added by Chabris and colleagues, 2015) variation across complex phenotypes is the product of numerous genes, all generally contributing very small effects.

By the time Polderman et al. (2015) published their recent meta-analysis of twin studies, thousands of quantitative genetic studies had been carried out across various academic fields, ranging from sociology to medicine. In an effort to synthesize this literature, Polderman and colleagues (2015) calculated average heritability estimates for a range of medical, psychological, and behavioral domains. The results, to summarize briefly, aligned with impressive fidelity with the laws proposed by Turkheimer (2000) nearly a decade and a half prior. In short, human variation across a range of behaviors, cognitive abilities, and physiological traits is influenced by genetic variation, as well as environmental variation, and in particular, idiosyncratic and unique environmental experiences that function to create phenotypic differences between siblings (the non-shared environment).

A final and important point worth considering at this juncture is that a trait maybe entirely biological in origin, yet also have a heritability of zero (Plomin et al., 2013). For instance, developing two eyes during gestation is a genetically encoded process in the human species. Any deviation from that species typical condition is likely due to some environmental event (Pinker, 2002; Barnes et al., 2014). Losing an eye in an accident, for example, means that one differs from other members of the population for number of functional eyes because of environmental factors, not genetic factors (Pinker, 2002). Assuming one rejects metaphysical explanations of consciousness, we seem compelled to embrace a biological explanation of consciousness that is rooted in brain function.

Yet, consciousness may vary, and clearly does vary in certain instances, purely because of environmental factors such as patients experiencing vegetative states in the wake of severe brain trauma or lesion (Parvizi and Damasio, 2003). If this is indeed the case in every instance when consciousness varies, then heritability estimates of consciousness (assuming we could calculate them) will be zero. In reality, this would only slightly alter the propositions outlined herein about consciousness. However, for reasons discussed below, it seems unlikely that heritable variation is irrelevant for creating variation in consciousness in the population. I attempt to wade slowly into the discussion, though, by first briefly mentioning work on the “neural correlates of consciousness.”

### 2.2 Neural correlates of consciousness

Neural imaging techniques have been used for some time to explore a range of topics in neuroscience, and it was only a matter of time before consciousness researchers pressed the techniques into service in order to examine aspects of consciousness. The result has been an increase in understanding about specific neurological regions that appear to be implicated in various aspects of conscious experience and awareness (Bor, 2012; Tononi and Koch, 2008, 2015; Crick and Koch, 2003; Block, 2005; Cohen and Dennett, 2011). It is not necessary to exhaustively list all of the brain regions linked with aspects of consciousness in order to make that point that lesioning certain—but not all—brain regions can limit the range of conscious experiences that an individual can experience (Bor, 2012; Parvizi and Damasio, 2003). As was discovered when Phineas Gauge experienced the neurological and physiological trauma of having an iron rod shot through his head, it is possible to still have a conscious, subjective experience, even if part of the frontal lobe is damaged or removed (Bor, 2012; Damasio, 1994).

To use a different example, if you temporarily alter functioning in the thalamus (for example) with common anesthetics, a patient will reach a point at which consciousness is lost (Tononi and Koch, 2008). As Tononi and Koch (2008) also point out, similar sites of import for anesthesia include posterior cingulate, medial parietal cortical areas, and medial basal forebrain (see Bor, 2012 for additional discussion). Parvizi and Damasio (2003) have also pointed out that lesions in the pons can alter conscious states—seemingly, removing consciousness entirely (Långsjö et al., 2012; Monti et al., 2010). The overall point is that some neural regions impact consciousness—and in these cases, often the “levels” of consciousness—in a more profound way than others, such that altering functioning in those areas results in a dramatic shift in experience (Långsjö et al., 2012; Monti et al., 2010). This does not mean, however, that altered functioning across other neural regions or structures fails to shift consciousness in less profound, but measurable ways. In many of these instances, moreover, it may in fact be the content of the conscious experiences that are more heavily impacted than the levels of the consciousness experienced by the individual.

In their discussion of NCCs (i.e., neurological correlates of consciousness) and their own overarching theory of consciousness (Integrated Information Theory), Tononi and Koch (2008) note that (p.255):

*“Naturally, the integrated information theory converges with other neurobiological frameworks (e.g.,**Crick and Koch, 2003**; Edelman, 1989; Dehaene et al., 2006) and cognitive theories (Baars, 1988) on certain key facts: that our own consciousness is generated by distributed thalamocortical networks, that reentrant interactions among multiple cortical regions are important, that the mechanisms of consciousness and attention overlap but are not the same, and that there are many “unconscious” neural systems.”*

The key implication here is that theories of consciousness have to account for a shared set of apparently well supported findings. Which, in this case includes regions of the brain—like thalamocortical networks—that appear to play at least some role in conscious experience. As an aside, a key point was made recently by Von Opstal and colleagues (Van Opstal et al., 2014), when examining the role of striatal dopamine and measures of visual consciousness. As they note, thalamocortical connectivity is influenced by dopamine, and their findings suggested that individual variation in dopamine appeared to be correlated with differences in visual consciousness. Put another way, individual variation seems already linked—to some extent—with variation in aspects of consciousness. The question now appears to be: what explains the variation?

### 2.3 Variability & the heritability of brain structure

As mentioned above, the specifics of putative NCCs is not as important as acknowledging that activity within certain aspects of the brain appear to be at least correlated with subjective experience—both levels and contents of consciousness. A key question, however, is whether variation across these NCCs—which either exists naturally or is induced by environmental influence (such as drug use)—matters for the subjective experience of the person. All human hearts pump blood; thus, the heritability of blood pumping is zero (see Plomin et al., 2013). Yet, there is variability in how well a population of hearts function, and part of that variation could be heritable (for similar examples see Plomin et al., 2013; Pinker, 2002). Similarly, we might assume that *all* human brains produce conscious experience at a certain level, and also populate that conscious awareness with experience (i.e., contents). In that case, the heritability of “having consciousness” at a human specific level would be zero.

However, if it is the case at all that variation in the structure and function of neural regions alters the nature of conscious experience (it's contents), we might reasonably say that aspects of consciousness could be heritable. This returns the current discussion to a point mentioned earlier, which is that there seems to be two issues in play, the issue of hertiabiltiy and levels of consciousness, and that of heritability and content of consciousness. Again, this is not to argue that the two questions are completely orthogonal, only that we might empirically arrive at one answer for one question, and a different answer for the other.

Peper et al. (2007) reviewed the research that has utilized both neuroimaging and twin techniques to examine the heritability of brain volume and the structure of specific brain regions and features (using measures such as cortical thickness). Various measures emerged as moderately to highly heritable (Anokhin et al., 2008). For instance, several studies revealed heritability estimates ranging from .7 to .9 for measures assessing grey matter, white matter, and total brain volume. Variation across other measures, such as the lateral ventricles, was explained a more by environmental factors. Importantly, individual differences existed for brain regions mentioned above which have been linked in various respects to conscious experience—such as the thalamus—with results also pointing toward a high similarity between MZ twins (*suggestive* of a genetic effect).

A limitation is that many of the studies included in the review did not examine large samples of twins, thus the results should be appreciated with that consideration in mind. Nonetheless, Peper et al. (2007) (p.471) noted that: “Taken together, MRI studies in twins indicate that, given the basic additive genetic model, overall brain volume in adulthood is highly heritable.” In other words, individual differences exist, and in some cases, are partly heritable, for a variety of brain regions that are associated with different aspects of perception, awareness, language, memory, and executive functioning (Simons and Chabris, 1999; Toga and Thompson, 2005; Thompson et al., 2001). It would seem reasonable, then, to argue that these individual differences may impact the types of conscious experiences of individual human beings, and perhaps variation in levels of conscious experience as well.

### 2.4 Variability and human experience

None of the points discussed above do damage to the idea that it is “like something” to be human, regardless of the cognitive hardware and how it may differ from person to person. Yet, as this review has argued, it can also be the case that the nature of the experience differs from person to person and is informed by individual differences. Consider as an example, what it is like to be schizophrenic. It is undoubtedly like something to have, and live with, schizophrenia (a highly heritable disorder; see (Sullivan et al., 2003). Nonetheless, it also seems possible that the nature of conscious experience differs from individuals with schizophrenia to those who lack the disorder (and moreover, likely differs from schizophrenic to schizophrenic). Another example that makes this same point is autism, a highly heritable quantitative trait (see Bor, 2012; and Lichtenstein et al., 2010) for an overview of autism). Individuals with autism can have rich and fulfilling emotional lives. It is “like something” to be alive, and to have autism. Yet, given the spectrum aspect that exists for autism disorders, we might suppose than an individual scoring high on the autism spectrum experiences a different type of consciousness (in terms of contents) than someone scoring lower (see Bor, 2012 for an extended discussion). Given the role that the genes play in locating individuals along the autism spectrum, it seems reasonable that this heritable variation informs the nature of the subjective experience.

### 2.5 Consciousness: continuous or dichotomous?

Operating in the background of this discussion, thus far, has been the question of whether conscious experiences represent hard dichotomies (i.e., conscious or not) or graded levels of experience. This becomes critical to consider in particular, when one attempts to quantify and measure conscious experiences. Experimental studies of consciousness rely on a variety of measures aimed at quantifying consciousness, in many cases so that researchers might attempt to isolate the involvement of putative NCCs (Overgaard et al., 2006; Sergent and Dehaene, 2004). The most important point to keep in mind is that the arguments herein do *not* depend on one particular measure being preferred over another, or even whether the studies that have utilized these measures are free from methodological limitations (indeed, some of them do have important shortcomings).

The attentional blink (AB) task, for example, is a putative measure of consciousness employed by researchers. Participants are asked to identify “target” images (e.g., four letter words). Target 1 is presented, followed by a series of “distractor images” and then Target 2 is made visible (Sergent and Dehaene, 2004). Sergent and Dehaene (2004; p.721) describe both the AB task more thoroughly, and their goal, thusly:

*“The AB is observed when two targets are embedded in a rapid sequence of distractors: Correct identification of the first target (T1) hinders explicit report of the second target (T2) if they are separated by 200 to 500 ms (Broadbent and Broadbent, 1987). The AB affects a vast range of explicit tasks on T2, but the behavioral measures currently used to detect the AB (accuracy on a forced-choice task) do not allow one to determine whether participants are really unconscious of that target, especially given that accuracy is often slightly above chance level. We examined whether the AB merely degrades the available information on T2 or corresponds to an all-or-none loss of conscious perception of T2. To this end, we asked participants to rate the visibility of T2 on a continuous scale.”*

Their analysis suggested that a dichotomy exists between conscious perception and no perception at all. Caution is warranted, however, from a methodological point of view in that the sample size was very small (10 participants), the extent that the findings replicate remains an empirical question.

Overgaard and colleagues (Overgaard et al., 2006) argued that for a variety of reasons, the interpretation of Sergent and Dehaene (2004) required revision. Using a continuous measure known as the Perceptual Awareness Scale (PAS), in which subjects are presented with a stimulus and asked to rate the clarity with which it is perceived (no experience; brief glimpse; almost clear experience; clear experience), results from a new set of experiments pointed toward a graded conception of consciousness as opposed to binary (Overgaard et al., 2006). As with prior work, it should be acknowledged that small sample sizes can adversely impact the results and the subsequent interpretation. The more important point is that measures have been developed to assess various aspects of conscious perception and awareness. The only requirement needed to test the arguments of the current paper, which is that variation in consciousness is partly heritable, is to utilize these measures in a sufficiently large sample of twins. To the extent that MZ twins perform more similarly than DZ twins, it will serve as some evidence of a genetic influence on conscioussness. To the extent that DZ twins perform about as similar to one another as do MZ twins, it will be evidence of an environmental effect (Plomin et al., 2013).

One might argue that the measures mentioned above are not measures of consciousness, but rather simply tap into aspects of perception or attention. This is a reasonable concern. From a basic psychometric point of view, the goal is to have reliable and valid measures that are consistently and accurately measuring what we think they are measuring. To be sure, measures like AB and PAS are measuring *something*, and the something that they are measuring may indeed be partially heritable, but it might simply be that they are just not measuring *consciousness* in ways that we would want to define the phenomenon. All that this suggests is that better measures are in need of being developed (more generally, see (Tononi and Koch, 2008).

To date, there has been some effort to measure various constituent components of consciousness using samples of siblings in order to decompose trait variance into heritable and environmental components. Norbury et al. (2007) examined sensitivity to pain using a variety of standard measurement protocols (e.g., thermal burn protocol). The pattern of results revealed greater similarity for MZ versus DZ twins regarding the experience of painful stimuli. Norbury and colleagues (Norbury et al., 2007), in particular, reported that a model of additive heritability and nonshared environment (the AE) fit the data best for most of the measures. Heritability estimates explained between 20 and 50 percent of the variance in most cases (see also (Mogil, 2012). Additionally, some emergent evidence suggests that variation in the experience of certain tastes and flavor profiles may also be partly heritable (Newcomb et al., 2012).

One might rightly contend that partly heritable experiences of pain, as well as the experience of taste, does not constitute dispositive evidence of the arguments presented herein. However, what these two examples do illustrate is that conscious experience has constituent parts that are measurable and amenable to behavioral genetic analysis. To the extent that we want to empirically test aspects of consciousness more broadly we will need to measure it. To the extent that we can measure it, we can expect that individual differences likely exist in performance on the measures. In other words, the ideas suggested here are plausibly testable and the variance in the population will be accounted for by some combination of heritability, shared environment, and non-shared environment.

### 2.6 Athleticism as analogue for consciousness

As a final way of illustrating the concepts of the current argument, consider athleticism as a final unifying example. While others may have used athleticism as an example to illustrate various concepts. To my knowledge, it has not yet been used to make this particular point about variability in consciousness. Athletic endeavors assume a variety of forms, and yet there are various factors that overlap whether one is playing tennis or basketball. For instance, well-coordinated fine and gross motor movement is usually imperative. Spatial awareness and the ability to gauge distance accurately are critical, whether one is shooting the basketball or putting a golf ball. All of these behaviors emerge from coordinated action across a variety of systems. Moreover, one could construct a unified understanding of athletic ability; yet doing so is not necessary for recognizing a clear reality about athleticism (however we might operationalize it). Individual differences exist across nearly all of the traits that are recruited in athletic performance. The reality of this fact is as simple as a recognition that not everyone can perform at the same level as professional athletes.

The sources of individual differences are important to some degree, as some of the individual differences will be the product of heritability while other sources of variance will be environmental in origin (i.e., owing to practice). Nonetheless, individual differences exist. At the same time, all humans (barring some serious developmental anomaly or environmentally induced trauma) are capable of “gross motor movement” and “fine motor movement” to the extent that they might shoot a basketball. Thus, the idea “that everyone is an athlete” to some important degree is true. Similarly, everyone is conscious. Yet, if what I am proposing is accurate to any extent, consciousness may be naturally variable and quantitative. And assuming you could measure it, you would find that at least some of the variation is likely to be heritable.

### 2.7 An argument from evolution

As I mentioned earlier, while it is not the focus of the current review, there is debate about whether consciousness is an adaptation fashioned by selection pressures because it conferred some reproductive advantage, versus whether it exists as a by-product of adaptation or a genetic accident (Miller, 1999). This topic does have some relevance for the current discussion, so it is worth spending a brief amount of time on the issue. Regardless of the position that one might stake out, consciousness arose somehow via the processes of evolution (genetic drift, selection, etc.). In order for natural selection to work, there must be genetic variation in a population of organisms, the result of which is non-random selection of genes that confer some fitness advantage (Williams, 1966).

As has already been argued, it seems clear that conscious awareness involves various neural regions which produce subjective awareness/experience (Bor, 2012; Långsjö et al., 2012; Parvizi and Damasio, 2003). In an evolutionary framework, then, to the extent that subjective experience (or the ability to have some version of it) conferred a fitness advantage for individuals in the population, evolutionary forces would operate to positively select genetic variation related to the neurological variation which helped produce the experiences. At a minimum, then, consciousness (or in particular, the brain regions that ultimately allow us to produce certain aspects of consciousness) was heritable in our species at some point in our evolutionary history (Bor, 2012).

### 2.8 Evolution & a brief word on attention schemas and awareness

Earlier, I alluded to the “hard problem of consciousness”, an idea articulated by Chalmers (1995) years ago. Chalmers (1995) bifurcates consciousness into two types of problems in need of solving; a hard and an easy problem. The easy problem is the one charged with identifying various neural structures implicated in conscious awareness, experience, and perception (i.e., the NCCs discussed above). The hard problem, in part, consists of trying to discern why (and to some extent, how) we have subjective experiences at all. For instance, it is possible for natural selection to design more rudimentary nervous systems, capable of achieving the imperatives of survival and reproduction, and yet (presumably) lacking in the rich subjective experiences that human beings report daily when they look at a painting or reminisce on fond memories.

With these points in mind, Graziano (2013; Webb and Graziano, 2015) proposed a “mechanistic” theory of consciousness called the attention schema theory (AST). What follows here is a highly abbreviated description of its basic arguments. AST draws on research about body schemas and the way in which brains construct working models to coordinate the movement and activity of our bodies. To utilize an example used by Webb and Graziano (2015; see p.5, Figure 2); imagine sitting at your desk with your right arm under the desktop so that you cannot see it. Assume now that several minutes have passed, and you're asked to maneuver your left hand to a position on the desk which is directly above your right hand. You can perform the task in general, but it is made more difficult by the fact that your brain is constructing a model of where your hand should be, without the benefit of visual information. Regarding the construction of models, Graziano and Webb (2014) further point out (p.3): “That model is constantly updated. It keeps track of body segments, their sizes, shapes, joint angles, speed, force, the tension on muscles, and other properties. The model can also help to make predictions a few seconds into the future.” In other words, it's a generally effective approach to operating and maneurvering the human body in space, though certainly not free from error.

Extending the idea of body schemas, Webb and Graziano (2015; p.9) note that: “The core claim of the theory is that the brain computes a simplified model of the process and current state of attention, and that the content of this model is the basis of subjective reports.” However, why would any of this matter for the current discussion of variability in consciousness? Returning quickly to body schemas, Graziano and Webb assert that (2014; p.7): “By analogy, when the brain lacks a clear internal model of the arm, the control of the arm is compromised.” The same argument, then, might also be applied to attention schemas, and the consequences when they break down. It is this point, in fact, that constitutes the most important reason to mention AST in the current context. Graziano (2013) seems to suggest that consciousness may represent a biological adaptation. He contends that humans construct internal models of themselves (of their awareness and attention), and of other people. In so doing, we attribute awareness and subjective experience not only to ourselves, but also to other people.

By attributing awareness and the capacity for attention to other people, we are equipped to anticipate their behaviors, thoughts, and feelings, something that should impact various key aspects of fitness, perhaps including finding a mate (Graziano and Kastner, 2011; see also, Miller, 1999). Consider scenarios when this ability seems interrupted or diminished, such as with individuals located on the autism spectrum (Lichtenstein et al., 2010). Typical theory of mind abilities become impaired, as do aspects of social processing (Graziano, 2013). As I mentioned earlier, variation across the autism spectrum is highly heritable, meaning that variation in the social impairment that can accompany autistic disorders is partly shaped by genetic variation. It is also worth mentioning, however, that variation on theory of mind measures—at least in early childhood for one prior study, seem primarily attributable to environmental factors (Hughes et al., 2005). Nonetheless, if Graziano is indeed correct about what subjective experience is, and why it exists, then it may further underscore the likelihood of heritable variation existing (at least at some point in our history) for human consciousness, as such variation would have been necessary for natural selection to operate.

## 3 Conclusion

Assuming any of the arguments presented above are correct, the key points seem to be as follows. Consciousness emerges via action across various brain regions. Brain regions involved in key components of consciousness, such as perception, attention, and awareness, all vary in the population, and are partly heritable. We know already that the content of conscious experiences, as well as levels of consciousness, can be varied via external factors such as psychedelic drugs, anesthesia, and brain trauma, and in principle it seems reasonable that genetic factors could also contribute to “trait-relevant” variation by altering brain structure and function during development and across the lifespan—thus producing different qualia. It seems possible to assume that the conscious experiences associated with certain psychopathologies like schizophrenia and autism are different than those experienced by humans without the disorder, or with a less severe version of the trait (see also Bor, 2012). As long as these propositions hold, we might assume that variation in consciousness exists, and may in some instances be partly (though not totally) the result of genetic variation.

### 3.1 Why should the heritability of consciousness matter?

On one hand, heritable variation seems to be the rule in nature, not the exception (Polderman et al., 2015). The putative heritability of consciousness may in fact, be like the heritability of height; something that exists, is interesting, but in the end provides little in the way of value or scientific insight. Yet, even height (which seems banal) provides a useful mechanism for testing a variety of scientific ideas, including examining recent selective pressures acting on human populations (Turchin et al., 2012). Unlike height, consciousness represents the root of our inner emotional lives, the experience of which seems essential to our daily existence, reflecting what it is “like to be us” (Nagel, 1974).

Relatedly, Hofstadter (2007) (using the word “soul” for consciousness and huneker to coin a unit of measurement) noted that (p.21);

*“There is an average tallness for adults, but there is also a considerable spread around that average. Why should there not likewise be an average degree of souledness for adults (100 hunekers, say), plus a wide range around that average, maybe (as for IQ) going as high as 150 or 200 hunekers in rare cases, and down to 50 or lower in others?”*

To the extent that Hofstadter (2007), and the arguments contained herein are correct, the implications seem large. Individual variability is tethered to virtually every topic social and natural scientists study, thus understanding variability in consciousness (should it exist) could shed insight on a host of important questions across a range of other fields (ranging from medicine to psychology). Equally important, examining if, and to what extent, measures of consciousness are heritable is a key piece of the puzzle for understanding, not only how consciousness evolved, but also how selection pressures might continue to act (or not act) on the human experience of consciousness (Miller, 1999; Hofstadter, 2007).

Ultimately, the propositions asserted herein are empirical and testable, but remain in need of actual testing. Nonetheless, there is already reasonably compelling evidence to support the contention that consciousness variation is partly heritable. If one is persuaded at all that consciousness arises from brain structure and function, and that we might be able to quantify and measure it, then we are compelled to at least entertain, and to test, the possibility of heritable variation. Whether, and to what extent, variability in consciousness is heritable in human beings remains an open and fascinating question.

## Declarations

### Author contribution statement

Brian Boutwell: Conceived and designed the experiments; Performed the experiments; Analyzed and interpreted the data; Contributed reagents, materials, analysis tools or data; Wrote the paper.

### Funding statement

This research did not receive any specific grant from funding agencies in the public, commercial, or not-for-profit sectors.

### Competing interest statement

The authors declare no conflict of interest.

### Additional information

No additional information is available for this paper.
